# Ribonucleotide reductase repression: a mutational study of NrdR-binding motifs

**DOI:** 10.1128/jb.00274-26

**Published:** 2026-08-27

**Authors:** Saher Shahid, Daniel Lundin, Inna Rozman Grinberg, Britt-Marie Sjöberg

**Affiliations:** 1 Department of Biochemistry and Biophysics, Stockholm University 7675, Stockholm, Sweden; National Institutes of Health, Bethesda, Maryland, USA

**Keywords:** binding site motifs, NrdR boxes, ribonucleotide reductase, NrdR, transcriptional repressor

## Abstract

The prevalent transcriptional repressor NrdR binds to highly conserved sequences in the promoter regions of prokaryotic operons encoding the essential enzyme ribonucleotide reductase. The NrdR-binding sites consist of two partially palindromic 16 bp sequences (NrdR boxes) separated by a 15–16 bp linker sequence. We have assessed the requirement of both boxes for binding, the propensity of different NrdRs to bind to heterologous binding sites, and that the linker sequence is only limited to length and not sequence conservation. As we have observed several deviations from the conserved sequences of the NrdR boxes, we have tested the conservation requirements of individual base pairs in the NrdR boxes using a synthetic DNA fragment (Synt DNA) to which the NrdR proteins from the actinomycete *Streptomyces coelicolor* and the gammaproteobacterium *Escherichia coli* bind equally well as to their homologous binding sites. By introducing isolated mutations to Synt DNA and testing the binding capacity of NrdR from *S. coelicolor* and *E. coli*, we expand our understanding of what criteria are needed to build a functional binding site for the NrdR repressor. Our results enable more precise identification of NrdR-binding sites across bacterial genomes, which can be used to explore NrdR and its regulons as potential targets for the development of novel antimicrobials.

**IMPORTANCE:**

The transcriptional repressor NrdR is strictly prokaryotic, occurring in 77% of bacterial genomes, and 22% of archaeal genomes. It binds to highly conserved sequences in the promoter regions of the genes encoding the essential enzyme ribonucleotide reductase that provides cells with *de novo* building blocks for DNA synthesis. Repression of ribonucleotide reductase expression leads to a lack of replication and cell proliferation. In this report, we have characterized in detail the generality of the partially palindromic sequences constituting the NrdR-binding site. As NrdR controls the sole *de novo* pathways for production of DNA building blocks in bacteria and not in eukaryotes, our results will facilitate screens for novel antimicrobials utilizing the NrdR protein and its homologous regulons in pathogenic bacteria.

## INTRODUCTION

The *nrdR* gene is found in a majority of bacterial genomes (1). It regulates expression of genes encoding the universal and essential ribonucleotide reductase (RNR) enzymes that provide the building blocks for DNA replication and repair in all cells (2–5). The NrdR repressor protein functions by binding to specific sites in the RNR-encoding promoter regions that overlap the RNA polymerase binding site (6–12). It has two binding sites for adenine nucleotides, an “inner” and an “outer” site. When ATP levels are high, both sites are occupied by ATP, and the NrdR protein adopts higher oligomeric forms that are unable to bind to DNA (10, 12). However, when dNTP levels increase as a result of halted DNA synthesis, ATP in the outer site is replaced by dATP, inducing a tetrameric form that binds to DNA and inhibits expression of RNR-encoding genes.

**Fig 1:**
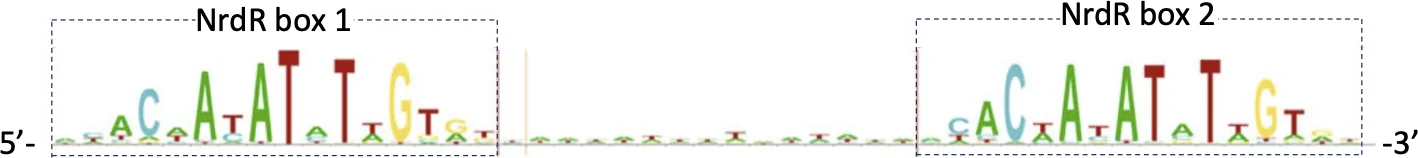
Logo of NrdR boxes from 935 sequences (10). Each NrdR box is a partially palindromic 16 bp sequence usually separated by a stretch of 15–16 non-conserved bp.

The NrdR-binding site consists of two—each partially palindromic—16 bp NrdR boxes (boxes 1 and 2) at a distance of 15–16 bp. The partially conserved palindromic sequence of an NrdR box was initially predicted from a small number of bacterial genomes (13). Later, a bioinformatic analysis of species-representative genomes revealed the consensus sequence for the NrdR boxes (Fig. 1), where some positions in each box are highly conserved (10). A mutational study in *Streptomyces coelicolor* demonstrated that C4 and G13 were crucial entities of the binding site (10), and recent *in vivo* experiments established that correct spacing between the two boxes is crucial for high-affinity binding (11).

NrdR’s function has primarily been experimentally studied in *Streptomyces coelicolor* with two RNR-encoding operons (*nrdAB* and *nrdRJ*), *Escherichia coli* with three RNR-encoding operons (*nrdAB*, *nrdHIEF*, and *nrdDG*), and *Pseudomonas aeruginosa* with three RNR-encoding genomes (*nrdAB*, *nrdJab*, and *nrdDG*) (6, 10, 12, 14, 15). Since each of these three genomes displays discrepancies from the consensus sequence of NrdR boxes in its NrdR-binding sites, we have performed an extended mutational study to understand which nucleotides in the NrdR boxes are critical for binding to DNA. In this study, the NrdR protein from *E. coli* (EcoNrdR) and *S. coelicolor* (ScoNrdR) were analyzed for their ability to bind to wild type and mutated NrdR boxes using microscale thermophoresis (MST).

## RESULTS AND DISCUSSION

### Heterologous binding of NrdR to NrdR boxes

To establish whether NrdR from *E. coli* (EcoNrdR) can bind *S. coelicolor* NrdR boxes and vice versa, we compared binding of the two NrdRs to all five promoter regions. Binding of *S. coelicolor* NrdR (ScoNrdR) to *E. coli* RNR promoters was comparable to binding to its own promoter regions and resulted in binding affinities between 56 and 169 nM (Table 1 and Fig. A1A). Similarly, EcoNrdR bound to *S. coelicolor* promoter regions with affinities comparable to affinities to its own promoter regions and had affinities of 126 and 347 nM (Table 1 and Fig. A1B). These results indicate that NrdR from one organism can bind to NrdR boxes from other organisms due to similarity between the boxes as well as the proteins. Earlier studies have, for instance, demonstrated a striking structural similarity of the DNA-binding tetramers of ScoNrdR and EcoNrdR (10, 12).

**TABLE 1:** Heterologous binding of ScoNrdR and EcoNrdR to RNR promoter regions

Promoter region	Box sequences (positions 1–16 and 32–47)	ScoNrdR *K*_*D*_ (nM)	EcoNrdR *K*_*D*_ (nM)
EconrdAB	-CCCCTATATATAGTGT-//-GTACTACAGGTAGTCT-	169 ± 63	113 ± 27^*a*^
EconrdEF	-TTGCTATATATTGTGT-//-CAACTACATCTAGTAT-	56 ± 17	138 ± 32^*a*^
EconrdDG	-GCACTATATATAGACT-//-ACCCAATATGTTGTAT-	58 ± 23	160 ± 37^*a*^
SconrdAB	-ACACAACATCTGGGGG-//-GCACAAGATGTATGCT-	142 ± 31^*b*^	347 ± 81
SconrdRJ	-TCCCCACATCTAGTGG-//-AGCCCACAAGTTGTGG-	51 ± 11^*b*^	126 ± 33

^*a*^ From reference 12.

^*b*^ From reference 10.

### Design of a synthetic NrdR-binding site

Initially, we intended to use short DNA fragments containing a single NrdR box with five flanking base pairs and to mutate the box nucleotides one by one to test how each individual base pair affects binding affinity. However, the NrdR protein requires two NrdR boxes to bind to DNA, as shown in Fig. A2 for the lack of binding of *E. coli* NrdR to isolated NrdR box 1 or NrdR box 2.

**Fig 2:**
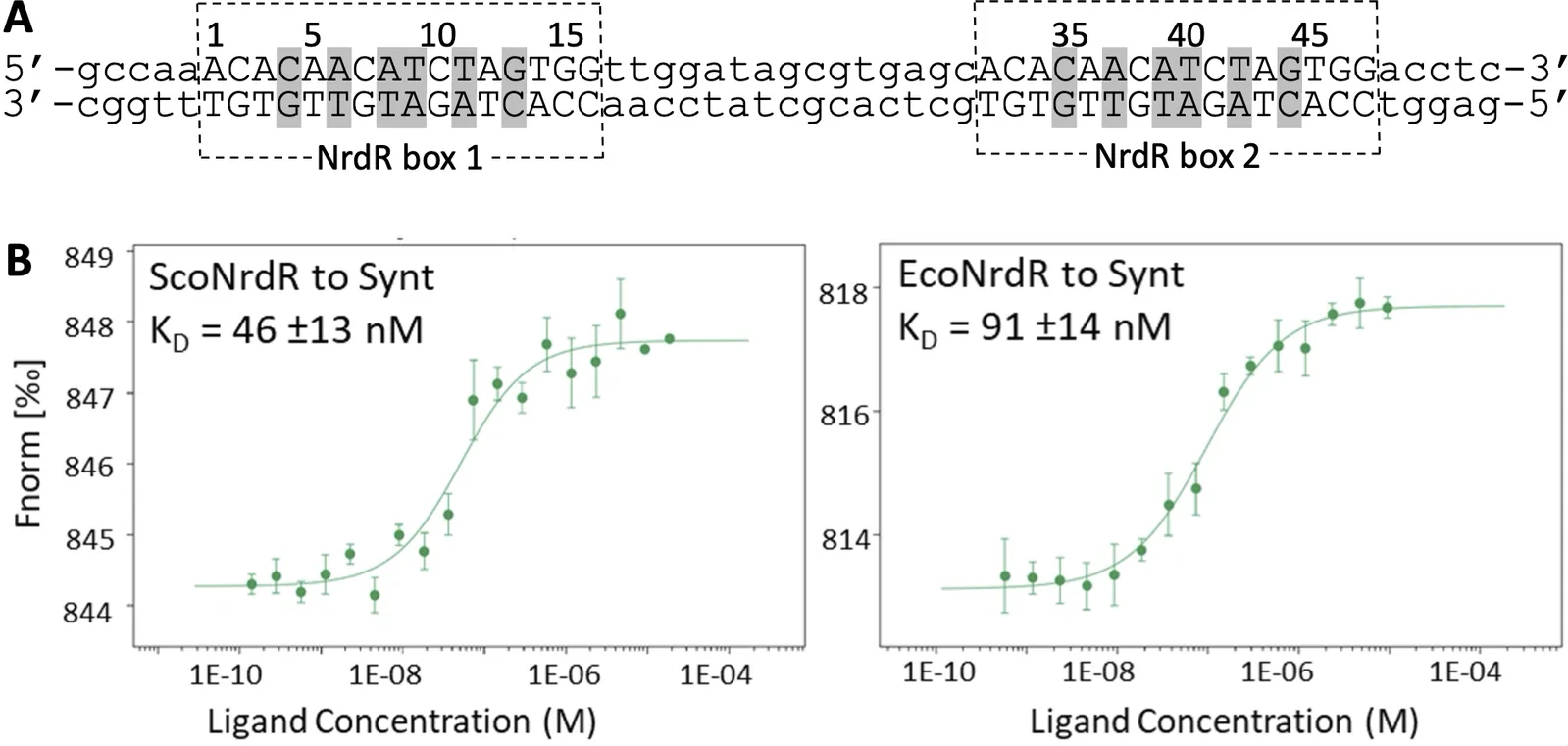
Binding of ScoNrdR and EcoNrdR to a synthetic NrdR box region based on the NrdR box logo sequence. (**A**) Sequence of a 57 bp synthetic DNA fragment (Synt DNA) with two identical NrdR boxes separated by a 15 bp linker sequence with numbering starting at NrdR box 1. NrdR boxes are in capital letters, highly conserved NrdR box positions shaded in gray, and flanking regions and the linker sequence in lowercase letters. (**B**) MST binding curves showing interaction of ScoNrdR and EcoNrdR to the 57 bp Synt DNA fragment. Data points represent an average of three independent experiments. *K*_*D*_ values were obtained by curve fitting using Nanotemper analysis software.

We therefore designed a synthetic NrdR-binding site denoted “Synt DNA,” consisting of two identical NrdR boxes connected via a 15 bp linker corresponding to the linker region of the *S. coelicolor nrdRJ* promoter (Fig. 2). The Synt DNA-binding site is flanked by 5 bp at each end. Both ScoNrdR and EcoNrdR had high affinity to the 57 bp Synt DNA with *K_D_*s comparable to binding to their wild-type promoters (Fig. 2 and Table 1).

The linker region between boxes 1 and 2 in natural binding sites is not conserved. Changing the linker region between the synthetic NrdR boxes from the *S. coelicolor nrdRJ* linker to the *S. coelicolor nrdAB* linker, which have only 13% identity (i.e., less than expected by chance), had only a marginal effect on the binding affinity of ScoNrdR or EcoNrdR (Table 2 and Fig. A3). This finding fits well with our earlier observation that NrdR proteins from four different species (*Listeria monocytogenes*, *Streptococcus pneumoniae*, *S. coelicolor*, and *E. coli*) had the capacity to bind to NrdR box pairs with linker regions corresponding to the predominant length of 15–16 bp, as well as a linker length of 26–27 bp, i.e., adding another DNA helix turn to the distance between NrdR boxes 1 and 2 (11).

**TABLE 2:** Effect of linker sequence on binding affinity of EcoNrdR and ScoNrdR

Promoter sequence	Linker sequences (positions 17–31)	ScoNrdR *K_D_* (nM)	EcoNrdR *K_D_* (nM)
Synt DNA + Sco*nrdRJ* linker	-TTGGATAGCGTGAGC-	46 ± 13	91 ± 14
Synt DNA + Sco*nrdAB* linker	-TGCTCGCGTCCCCCG-	32 ± 11	61 ± 15

### Mutations affecting highly conserved NrdR box positions

The consensus NrdR box is a partially palindromic 16 bp sequence in which six positions are highly conserved: C4, A6, A8, T9, T11, and G13 (Fig. 1). We previously showed that C4 and G13 in both NrdR boxes are critical for ScoNrdR binding to its native DNA sequence, where mutation of these nucleotides resulted in significant loss of affinity (10). To confirm this for EcoNrdR, we introduced the single mutation G44C in the native *E. coli nrdEF* NrdR box 2, which completely abolished binding (Table 3 and Fig. A4B). On the other hand, a comparative study of NrdR boxes in six different *Mycobacteriaceae* species predicted that box 1 in the promoter region of the *nrdHIE* operon had a G in position 4, whereas box 1 had a C4 in the promoter region of the *nrdF2* gene (16). We are not aware of any experimental studies showing that these sites bind NrdR.

**TABLE 3:** *K*_*D*_ values for binding of ScoNrdR and EcoNrdR to Synt DNA fragments and RNR promoter regions carrying mutations in conserved RNR box regions^*b*^^,^^*c*^

NrdR box mutations	Box sequences (positions 1-16 & 32-47)	ScoNrdR K_D_ (nM)	EcoNrdR K_D_ (nM)
Synt DNA wt^*a*^	-ACACAACATCTAGTGG-//-ACACAACATCTAGTGG-	46 ± 13	91 ± 14
Synt DNA G44T,T45G	-ACACAACATCTAGTGG-//-ACACAACATCTA**TG**GG-	56 ± 14	72 ± 19
Synt DNA A3C,C4A	-AC**CA**AACATCTAGTGG-//-ACACAACATCTAGTGG-	114 ± 21	206 ± 26
Synt DNA A3C,C4A, G44T,T45G	-AC**CA**AACATCTAGTGG-//-ACACAACATCTA**TG**GG-	383 ± 34	2,851 ± 502
SconrdAB T44G,G45T	-ACACAACATCTGGGGG-//-GCACAAGATGTA**GT**CT-	51 ± 28	n.d.
EconrdEF G44C	-TTGCTATATATTGTGT-//-CAACTACATCTA**C**TAT-	n.d.	>10,000
EconrdEF T12G,G13T	-TTGCTATATAT**GT**TGT-//-CAACTACATCTAGTAT-	n.d.	>12,000
Synt DNA A6C,A37C	-ACACA**C**CATCTAGTGG-//-ACACA**C**CATCTAGTGG-	1,115 ± 242	2,016 ± 336
Synt DNA A6C,T11G, A37C,T42G	-ACACA**C**CATC**G**AGTGG-//-ACACA**C**CATC**G**AGTGG-	No binding	No binding
Synt DNA A6G,A37G	-ACACA**G**CATCTAGTGG-//-ACACA**G**CATCTAGTGG-	167 ± 38	944 ± 161
Synt DNA A6T,A37T	-ACACA**T**CATCTAGTGG-//-ACACA**T**CATCTAGTGG-	138 ± 28	112 ± 30
EconrdAB box 0 + 1	-TCACACTATCTTGCAG-//-CCCCTATATATAGTGT-	n.d.	6,892 ± 1 017
EconrdAB box 0 + 1 C6A	-TC**A**CAATATCTTGCAG-//-CCCCTATATATAGTGT-	n.d.	598 ± 59

^*a*^ From Fig. 2A and Table 2.

^*b*^ n.d., not determined.

^*c*^ Bold letters in box sequences indicate mutated nucleotides.

The distance between C and G is usually 8 bp, but the *S. coelicolor nrdAB* promoter has a distance of 9 bp (Table 1). We investigated whether these conserved Cs and/or Gs could be moved one position in box 1, box 2, or both boxes of the Synt DNA sequence such that there are 9 bp between C and G. Mutations G44T plus T45G in box 2 resulted in high-affinity binding of both EcoNrdR and ScoNrdR (Table 3 and Fig. A4). The same was true when C4 was moved one position with mutations A3C plus C4A in box 1 of the Synt DNA sequence. However, moving C4 and G44 at the same time (A3C, C4A; G44T, T45G), so that there was 9 bp distance between the conserved C and G in both boxes, resulted in significant loss of binding (Table 3 and Fig. A4). Interestingly, reducing the distance between C and G in the *S. coelicolor nrdAB* promoter to 8 bp (T44G, G45T) resulted in a threefold tighter ScoNrdR binding, yielding an affinity similar to that observed for the *S. coelicolor nrdRJ* promoter (Table 3 and Fig. A4). To explore whether a 7 bp distance is acceptable between C and G in an NrdR box, we mutated the first NrdR box in the *E. coli nrdEF* promoter (T12G, G13T). This mutant lost the ability to bind NrdR (Table 3 and Fig. A4B), suggesting that reducing the spacing between the conserved C and G results in loss of NrdR binding. However, contributions from the accompanying sequence changes cannot be excluded.

A6 and T11 are highly conserved. Mutating A6 to C in both NrdR boxes (A6C plus A37C) resulted in more than 20-fold decrease in binding affinity for both EcoNrdR and ScoNrdR (Table 3 and Fig. A5). Moreover, mutation of both A6 and T11 in both NrdR boxes (A6C, T11G, A37C, and T42G) abolished binding completely (Table 3 and Fig. A5). For EcoNrdR, mutating A6 to G in both NrdR boxes decreased binding affinity 10-fold, whereas binding of ScoNrdR was decreased 4-fold (Table 3 and Fig. A5), suggesting that a GC base pair at this position significantly impaired NrdR binding. On the other hand, mutating A6 to T in both boxes did not have an effect on EcoNrdR binding, but still decreased ScoNrdR binding threefold (Table 3 and Fig. A5).

Earlier studies have annotated a different box pair in the *E. coli nrdAB* promoter consisting of box 1 (previously referred to as box 2) and an upstream box (previously referred to as box 1) covering the −10 region and the transcriptional start site (13, 17). However, we showed recently that this upstream box, denoted box 0 by us, is not recognized by EcoNrdR, and the NrdR-binding site in the *E. coli nrdAB* promoter consists of box 1 (previously referred to as box 2) and a functional NrdR box 15 bp downstream (12). To test if the lack of binding to the non-functional box 0 in the *E. coli nrdAB* promoter region is that position 6 in this case is a C instead of A, we introduced the mutation C6A in box 0 in Eco*nrdAB*, and this change resulted in 10 times higher affinity of EcoNrdR compared to Eco*nrdAB* wt (Table 3 and Fig. A5).

### The conserved middle AT sequence

**Fig 3:**
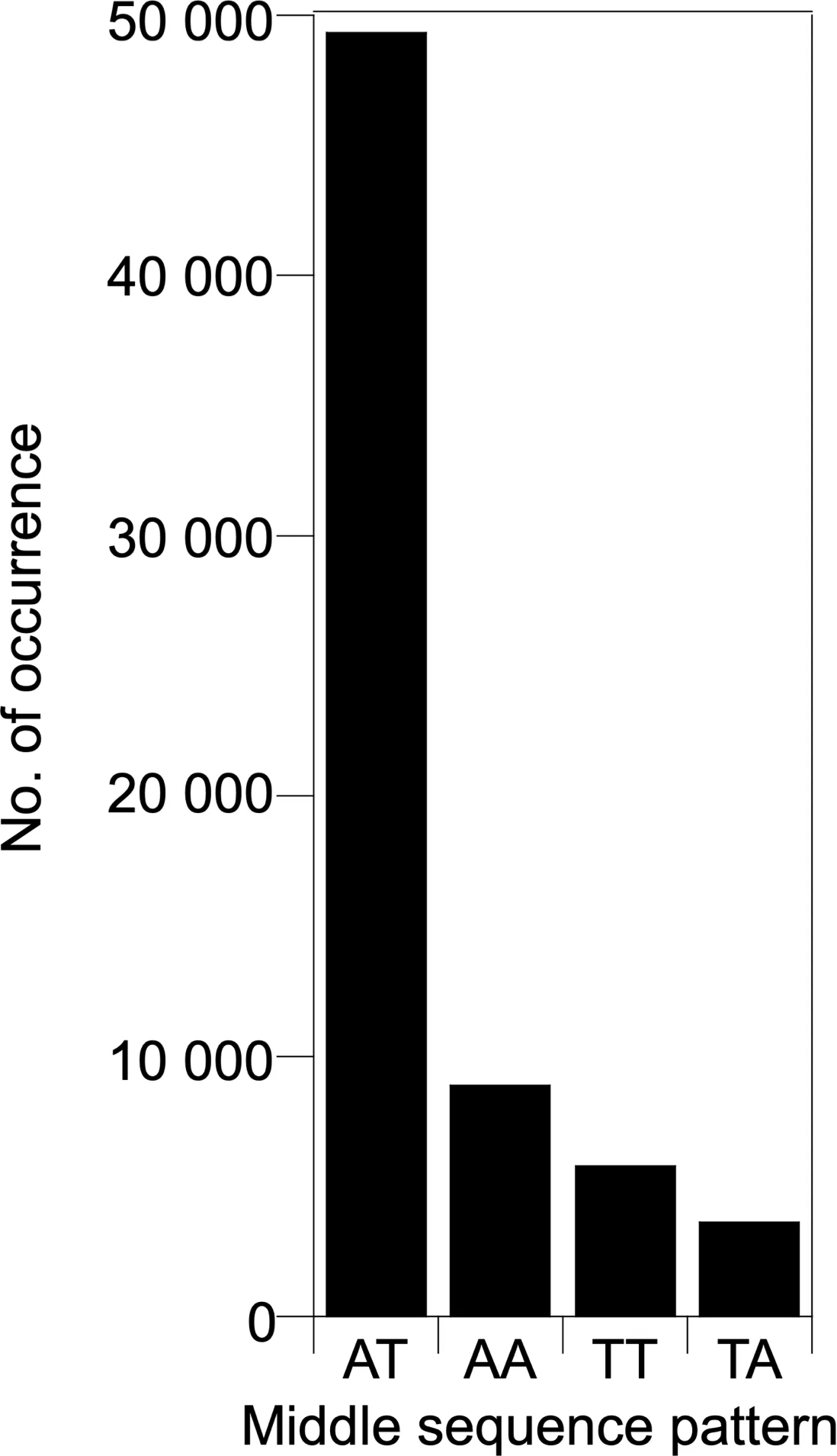
Frequencies of middle dinucleotide sequence in NrdR boxes.

The consensus NrdR box sequence predicts a middle conserved AT sequence (10, 13). Yet, box 2 in the *S. coelicolor nrdRJ* promoter region has a middle AA sequence, and box 2 in the *E. coli nrdAB* promoter region has a middle AG sequence (Table 1). We therefore performed a bioinformatic analysis of close to 60,000 NrdR-binding motifs in species-representative genomes from all bacterial species, and as expected, found that a middle AT sequence is predominant (Fig. 3). A middle AA sequence is six times less frequent, and middle TT and middle TA sequences are even less frequent.

High-resolution structures of ScoNrdR and EcoNrdR bound to DNA show that the dsDNA helix adopts sharp bends at each NrdR box (10, 12). This was primarily ascribed to the predominant middle AT sequence of the NrdR box. As both bioinformatics and *in vivo* observations (Fig. 3) (6, 10, 12, 14, 15) show that a middle AT sequence is not absolutely required, we checked the total AT content in the central part of NrdR box sequences in a few bacterial species with different genomic GC contents. The AT content between the conserved positions C4 and G13 varied from 63% to 100% in native NrdR boxes (Table 4). Although NrdR boxes from low-GC genomes generally showed higher AT content, this trend was not strict, suggesting that factors other than genomic GC content contribute to maintaining the AT-rich character of this region.

**TABLE 4:** AT content in NrdR boxes between the conserved positions C4 and G13 for different RNR promoter regions of bacterial species with different genomic GC content

Species	GC content (%)	Fraction AT base pairs			
		*nrdAB*	*nrdEF*	*nrdJ*	*nrdDG*
*Listeria monocytogenes^a^*	38	0.88; 0.94			0.88
*Streptococcus thermophilus*	39.1		1.0		1.0
*Streptococcus pneumoniae*	39.7		0.96		0.88
*Chlamydia trachomatis*	41.3	0.88			
*Escherichia coli^b^*	50.8	(0.88), 0.81	0.88		0.94
*Salmonella typhimurium*	52.2	0.88	0.75		0.88
*Pseudomonas aeruginosa^c^*	65–67	0.88		0.81	0.75
*Thermotoga maritima^d^*	67–69			0.79	1.0
*Streptomyces coelicolor^e^*	72	0.71		0.63	

^*a*^ Lm encodes two pairs of NrdR boxes 113 bp apart in the *nrdAB* promoter region.

^*b*^ Ec MG1655 encodes an NrdR box 0 in the *nrdAB* promoter region, which does not bind NrdR.

^*c*^ The Pa genome encodes a split *nrdJ* denoted *nrdJab.*

^*d*^ The Tm *nrdJ* promoter region has three NrdR boxes with a 14 bp linker in both cases.

^*e*^ The second NrdR box in the *S. coelicolor nrdAB* promoter region has a T in position 13 (instead of G) and a G in position 14.

We used our Synt DNA to experimentally check the importance of conserved A8 and T9. The T9A and T40A mutations, resulting in a middle AA sequence in one or both NrdR boxes, respectively, and A8T and A39T mutations, resulting in a middle TT sequence in both boxes, still had preserved binding affinity (Table 5 and Fig. A6). As the *S. coelicolor nrdRJ* promoter NrdR box 2 contains a middle AA sequence, we tested whether ScoNrdR would tolerate a middle AA sequence also in box 1 and introduced a T9A mutation in its natural NrdR-binding site. Sco*nrdRJ* T9A had a comparable *K_D_* of 83 nM to the *K_D_* of 51 nM for ScoNrdR binding to its natural Sco*nrdRJ* binding site, showing that ScoNrdR can recognize a binding site with a middle AA sequence in both NrdR boxes (Table 5 and Fig. A6A). Given the prevalence of a middle AT sequence (Fig. 3), the mutations A8C plus T9G in both NrdR boxes in Synt DNA may seem drastic, but had only a marginal effect on binding affinity for both ScoNrdR and EcoNrdR (Table 5 and Fig. A6). To confirm this surprising result, we also introduced an A39C mutation in the natural NrdR-binding site of the *E. coli nrdAB* promoter region, which has a middle AG sequence in box 2, changing its box 2 to a middle CG sequence. EcoNrdR binds to the Eco*nrdAB* A39C NrdR-binding site with similar affinity, *K_D_* of 127 nM compared to *K_D_* of 113 nM (Table 5 and Fig. A6B). So far, both our bioinformatic study (Fig. 3) and our experiments (Table 5), partly based on natural deviations from the predominant middle AT sequence in NrdR boxes, show that other middle dinucleotide combinations can be tolerated. The surprising discrepancy between the consensus box sequence and our finding that a middle AT sequence is not necessarily required conceivably relates to the fact that the rare occurrences of other base pairs in positions 8 and 9 are compensated by adjacent high content of AT base pairs, still allowing the sharp bend of DNA required for binding of the NrdR protein (10, 12). The strong conservation of the middle AT dinucleotide may also contribute to DNA flexibility or other promoter functions that are not directly reflected in our binding assays.

**TABLE 5:** *K*_*D*_ values for binding of ScoNrdR and EcoNrdR to Synt DNA fragments and mutated RNR promoter regions with altered AT content in the central region of the NrdR box^*b*^^,^^*c*^

NrdR box mutations	Box sequences (positions 1–16 and 32–47)	ScoNrdR *K*_*D*_ (nM)	EcoNrdR *K*_*D*_ (nM)
Synt DNA wt^*a*^	-ACACAACATCTAGTGG-//-ACACAACATCTAGTGG-	46 ± 13	91 ± 14
Synt DNA T40A	-ACACAACATCTAGTGG-//-ACACAACA**A**CTAGTGG-	57 ± 14	63 ± 12
Synt DNA T9A,T40A	-ACACAACA**A**CTAGTGG-//-ACACAACA**A**CTAGTGG-	41 ± 9	53 ± 9
Synt DNA A8T, A39T	ACACAAC**T**TCTAGTGG-//-ACACAAC**T**TCTAGTGG	36 ± 9	32 ± 8
SconrdRJ T9A	-TCCCCACA**A**CTAGTGG-//-AGCCCACAAGTTGTGG-	83 ± 10	n.d.
Synt DNA A8C,T9G,A39C,T40G	-ACACAAC**CG**CTAGTGG-//-ACACAAC**CG**CTAGTGG-	82 ± 17	65 ± 20
EconrdAB A39C	-CCCCTATATATAGTGT-//-GTACTAC**C**GGTAGTCT-	n.d.	127 ± 23

^*a*^ from Fig. 2A and Table 2.

^*b*^ n.d., not determined.

^*c*^ Bold letters in box sequences indicate mutated nucleotides.

### Mutations affecting non-conserved NrdR box positions

Mutating A5 to C in both boxes (A5C plus A36C) had no effect on binding affinity (Table 6 and Fig. A7). However, introducing mutations in both A5 and A12 in both boxes (A5C, A12G, A36C, and A43G) resulted in a binding affinity that was 10 times lower for ScoNrdR and 5 times lower for EcoNrdR, which is plausibly an effect of the resulting low AT content between C4 and G13. The native *S. coelicolor nrdRJ* promoter contains C in position 5 in both NrdR boxes, but either A or T in position 12. Mutation of A12 to G plus T43 to G in the *S. coelicolor nrdRJ* promoter resulted in 10 times decreased affinity (Fig. A7A), confirming the results with the Synt DNA.

**TABLE 6:** *K*_*D*_ values for binding of ScoNrdR and EcoNrdR to Synt DNA fragments and *S. coelicolor* RNR promoter regions carrying mutations in non-conserved NrdR box positions^*b*^^,^^*c*^

NrdR box mutations	Box sequences (positions 1–16 and 32–47)	ScoNrdR *K*_*D*_ (nM)	EcoNrdR *K*_*D*_ (nM)
Synt DNA wt^*a*^	-ACACAACATCTAGTGG-//-ACACAACATCTAGTGG-	46 ± 13	91 ± 14
Synt DNA A5C,A36C	-ACAC**C**ACATCTAGTGG-//-ACAC**C**ACATCTAGTGG-	62 ± 12	93 ± 21
Synt DNA A5C,A12G,A36C,A43G	-ACAC**C**ACATCT**G**GTGG-//-ACAC**C**ACATCT**G**GTGG-	447 ± 106	495 ± 107
SconrdRJ A12G,T43G	-TCCCCACATCT**G**GTGG-//-AGCCCACAAGT**G**GTGG-	597 ± 121	n.d.
Synt DNA C7T,C10A,C38T,C41A	-ACACAA**T**ATATAGTGG-//-ACACAATATATAGTGG-	45 ± 10	41 ± 15
Synt DNA A3C,A34C	-AC**C**CAACATCTAGTGG-//-AC**C**CAACATCTAGTGG-	46 ± 8	37 ± 10
Synt DNA T14G,T45G	-ACACAACATCTAG**G**GG-//-ACACAACATCTAG**G**GG-	46 ± 11	53 ± 10
Synt DNA A3C,T14G,A34C,T45G	-AC**C**CAACATCTAG**G**GG-//-AC**C**CAACATCTAG**G**GG-	33 ± 7	95 ± 12

^*a*^ From Fig. 2A and Table 2.

^*b*^ n.d., not determined.

^*c*^ Bold letters in box sequences indicate mutated nucleotides.

Mutating the non-conserved C7 and C10 in both NrdR boxes (C7A, C10T, C38T, and C41A) did not affect the binding affinity of either ScoNrdR or EcoNrdR (Table 6 and Fig. A8). Moreover, a duplication of *E. coli nrdEF* box 1 (with A7 and T10) or box 2 (with C38 and C41) had no effect on binding affinity (11), corroborating that positions 7 and 10 in the NrdR boxes are not conserved.

Mutations in position 3 and 14 toward the edges of the boxes, separately and together, had as expected very little effect on affinity (Table 6 and Fig. A9). These results exclude the possibility that a C and/or G next to C3 and/or G13 (as exists in *S. coelicolor* promoters) improves recognition of the box by NrdR.

### Concluding remarks

**Fig 4:**
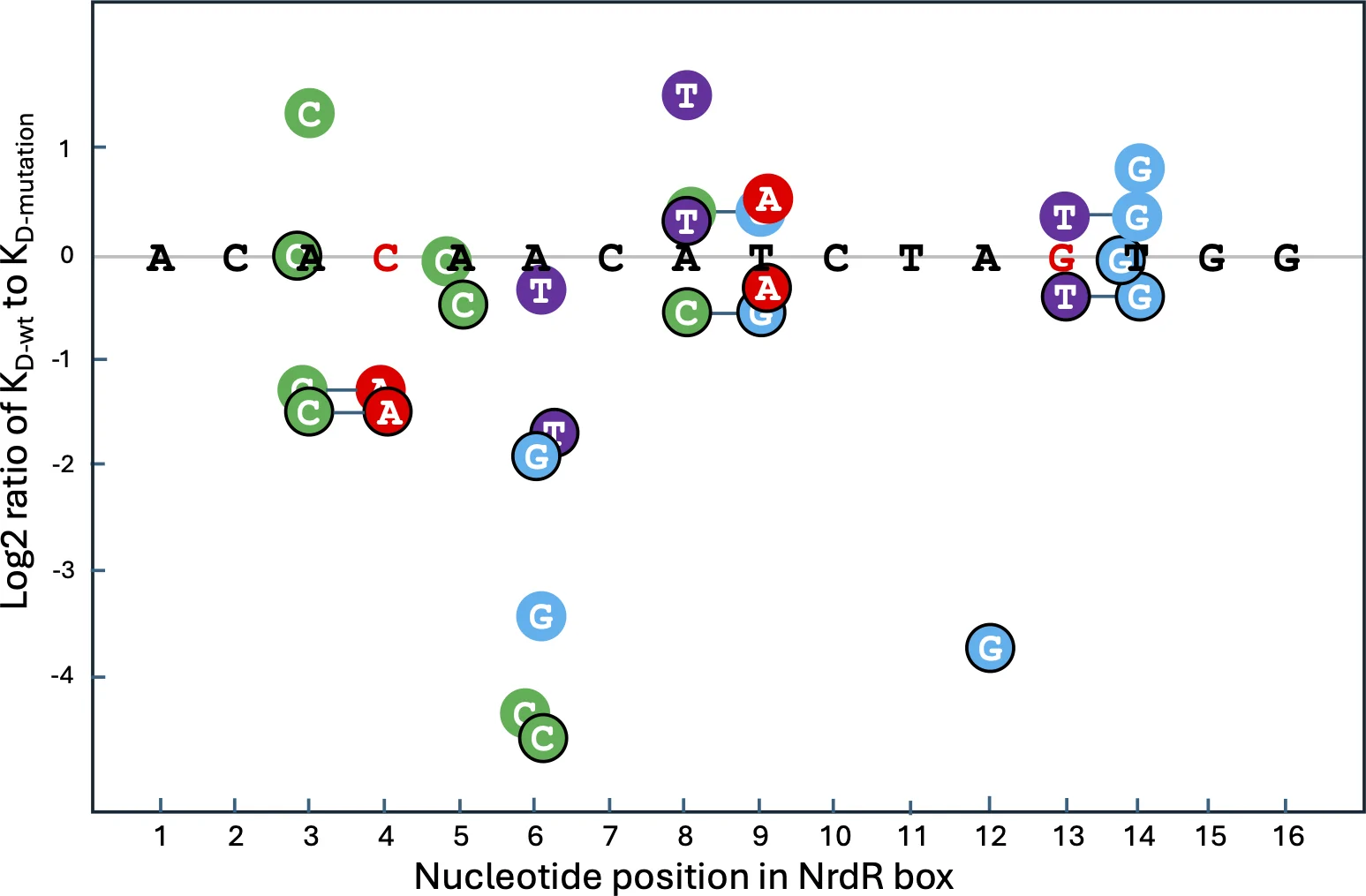
Summary of single and double mutation effects on binding of EcoNrdR and ScoNrdR to Synt DNA fragments. The Synt DNA sequence is shown in black, with essential C4 and G13 in red. Mutation effects with black contours refer to ScoNrdR binding, and without contours to EcoNrdR binding. Double mutations are connected via a bar. Positive log2 values indicate mutations with stronger binding than wt, and negative log2 values mutations with worse binding compared to the non-mutated Synt DNA. Mutation values close to wt values are slightly offset from their sequence positions for increased clarity.

In this work, we performed a systematic mutational analysis of NrdR boxes and demonstrated how specific alterations influence the binding strength of *S. coelicolor* and *E. coli* NrdR proteins. The key findings are summarized schematically in Fig. 4.

A bioinformatic study of species-representative genomes shows six highly conserved positions in each of the two NrdR boxes (Fig. 1), constituting the binding site for the prevalent prokaryotic transcriptional repressor NrdR (10). At the same time, part of the NrdR-binding site is also a binding site for sigma factors, RNA transcriptase subunits, and/or other regulatory factors, which may explain why some deviations from these conserved positions occur. Yet, the overall ability of NrdR to recognize its binding sites is preserved, suggesting that NrdR-binding sites evolved via a combination of some key conserved residues and DNA flexibility and spacing. Based on our mutation study of the NrdR boxes, we can now summarize the following observations:

- C4 and G13 are critical, and we showed earlier that these bases bind to residues Asp15 and Arg17 in *S. coelicolor* NrdR (10), but a distance of 9 bp rather than 8 bp between C and G is tolerated in one box but not in both.
- A6 and T11 seem critical, but no specific interaction with the NrdR protein has been identified. Plausibly, the partially palindromic structure of 6 and 11 is important *in vivo*.
- A8 and T9 are not absolutely critical for binding. Rather, there is a lower limit of 63% AT base pairs needed between positions C4 and G13 to allow for the sharp bend of dsDNA in the palindromic NrdR box.

Our results suggest that NrdR recognizes DNA through a combination of specific base interactions and overall shape and spacing of the DNA. This study will enable more precise identification of NrdR-binding sites across bacterial genomes to support the exploration of NrdR and its RNR regulons as potential targets for antimicrobial development in clinically relevant pathogens. A more precise understanding of NrdR-binding-site requirements may support future antimicrobial research by improving the identification of functional NrdR regulons in pathogenic bacteria. Since NrdR controls the expression of ribonucleotide reductases, which are essential for DNA precursor synthesis, disruption of NrdR-mediated regulation could interfere with balanced dNTP production, DNA replication, and bacterial proliferation. Thus, defining the DNA features required for NrdR binding may help identify bacterial species and regulatory sites suitable for future screens targeting NrdR-dependent RNR regulation.

## MATERIALS AND METHODS

### Bioinformatic analysis

After identifying potential RNR operons—genes annotated as RNRs by Prokka (v. 1.14.6 [18]), separated by no more than 300 nucleotides—in all species-representative bacterial genomes in the GTDB (release R08-RS214 [19]), 500 nucleotides upstream of each operon were extracted. Each fragment was searched with a regular expression for a pair of NrdR boxes separated by a spacer of no more than 50 nucleotides (“C.[AT].[AT][AT].[AT].G.{1,50}?C.[AT].[AT][AT].[AT].G”). Included in the search were sequences matching spacers with 20–23 nucleotides (corresponding to a box distance of 14–17 bp; 25,934 occurrences) and spacers with 30–33 nucleotides (corresponding to a box distance of 24–27 bp; 7,218 occurrences).

### Synthetic sequence and mutants

Cy5-labeled sense strands and unlabelled antisense strands were generally 57 bp long and purchased from Sigma-Aldrich. To elucidate binding of NrdR to individual boxes, shorter oligonucleotides of 26 bases were ordered, containing either individual NrdR box 1 of *nrdEF* RNR promoter or box 2 of *nrdEF* RNR promoter (Table A1). For simplicity, only Synt DNA wt is shown (Table A1).

### Plasmids and reagents

The *nrdR* gene from *S. coelicolor* strain M145 and from *E. coli* K-12 strain MG1655 were cloned into the pET30a(+) vector (Novagen; Merck KGaA, Darmstadt, Germany) as described previously (6, 17), resulting in pET30a(+)::Sco*nrdR* and pET30a(+)::Eco*nrdR* constructs producing the NrdR proteins with C-terminal hexahistidine (His-) tags. ATP and dATP solutions were purchased from Thermo Fisher Scientific.

### Production and purification of NrdR proteins

Production of ScoNrdR and EcoNrdR was performed as previously described for the respective proteins (10, 12), except that expression of the protein-encoding genes was induced at A_600_ 0.8–0.9 using 0.5 mM isopropyl β-D-1-thiogalactopyranoside.

ScoNrdR was purified as previously described (10), with some modifications. Briefly, the cell pellet was resuspended in lysis buffer containing 50 mM Tris-HCl, pH 8.0 at 4°C, 300 mM NaCl, 10% glycerol, 10 mM imidazole, and 2 mM DTT, whereafter 1 mM phenylmethylsulfonyl fluoride was added, and the cells were disrupted by sonication. The clarified lysate was applied to a HisTrap FF column (Cytiva), washed with buffers containing 10 mM and 60 mM imidazole, and eluted with 500 mM imidazole. The purified protein was desalted into storage buffer containing 50 mM Tris-HCl, pH 8.0 at 4°C, 300 mM NaCl, 10% glycerol, and 1 mM Tris(2-carboxyethyl)phosphine hydrochloride (TCEP) using HiPrep 26/10 Desalting column (Cytiva), and stored at −80°C.

EcoNrdR was purified according to the procedure described in reference 12. The storage buffer contained 50 mM Tris-HCl, pH 8.5 at 4°C, 300 mM NaCl, and 1 mM TCEP.

### Microscale thermophoresis

MST was used to study the binding of NrdR to native and mutant synthetic NrdR boxes. It measures changes in the movement of fluorescently labeled molecules in response to a temperature gradient. Binding of NrdR to Cy5-labeled DNA changes this movement, allowing dissociation constants (*K*_*D*_) to be determined from concentration-dependent binding curves, where normalized fluorescence (Fnorm, expressed in per mille, ‰) is plotted as a function of protein concentration.

MST was performed according to the procedure described in reference 11. Briefly, oligonucleotides of 57–58 bp containing two NrdR boxes separated by 15–16 bp and with flanking regions of 5 bp were annealed to obtain 1 µM dsDNA. The integrity of the annealed oligonucleotides was confirmed, and the DNA gel image is presented in Fig. A10. Binding of EcoNrdR and ScoNrdR to native and mutant DNA fragments was then analyzed by MST.

## SUPPLEMENTAL MATERIAL

Supplemental material jb.00274-26-s0001.pdf Fig. A1 to A10; Table A1.

ASM does not own the copyrights to Supplemental Material that may be linked to, or accessed through, an article. The authors have granted ASM a non-exclusive, world-wide license to publish the Supplemental Material files. Please contact the corresponding author directly for reuse.
