# Supplementary material for: Ribonucleotide reductase repression: a mutational study of NrdR-binding motifs
Source: J Bacteriol. 2026 Aug 27;208(9):e00274-26. doi: 10.1128/jb.00274-26 (PMC13584703; doi:10.1128/jb.00274-26)
Supplement: Supplementary file 1 [file jb.00274-26-s0001.pdf]

Appendixes

Ribonucleotide reductase repression: a mutational study of NrdR-binding motifs

Saher Shahid, Daniel Lundin, Inna Rozman Grinberg, Britt-Marie Sjöberg
Department of Biochemistry and Biophysics, Stockholm University, Sweden.

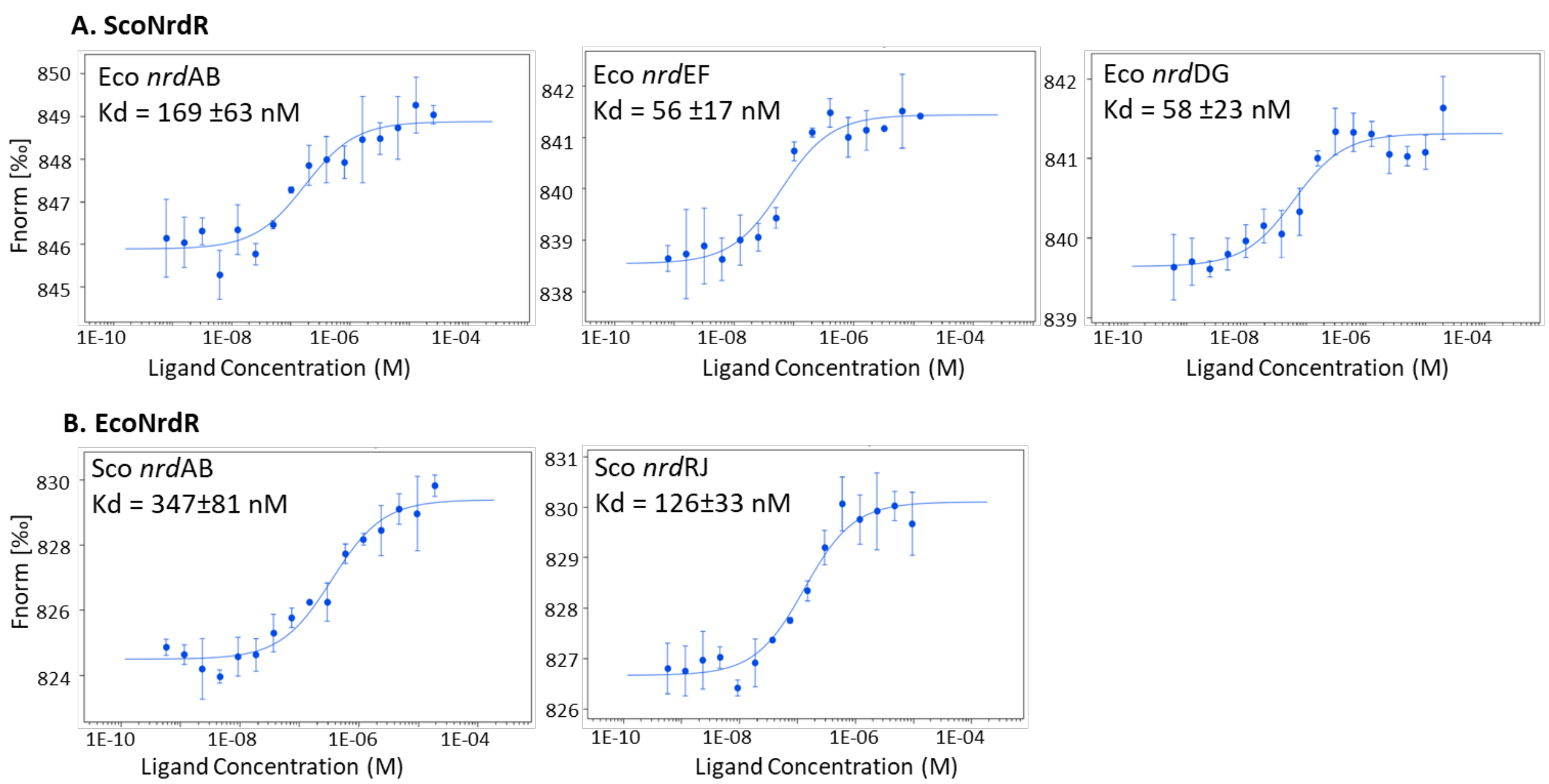

Figure A1. Heterologous binding of NrdR to RNR promoter regions. A. ScoNrdR binding to three *E. coli* RNR promoter regions, B. EcoNrdR binding to two *S. coelicolor* RNR promoter regions. All K_D values shown in the table above.

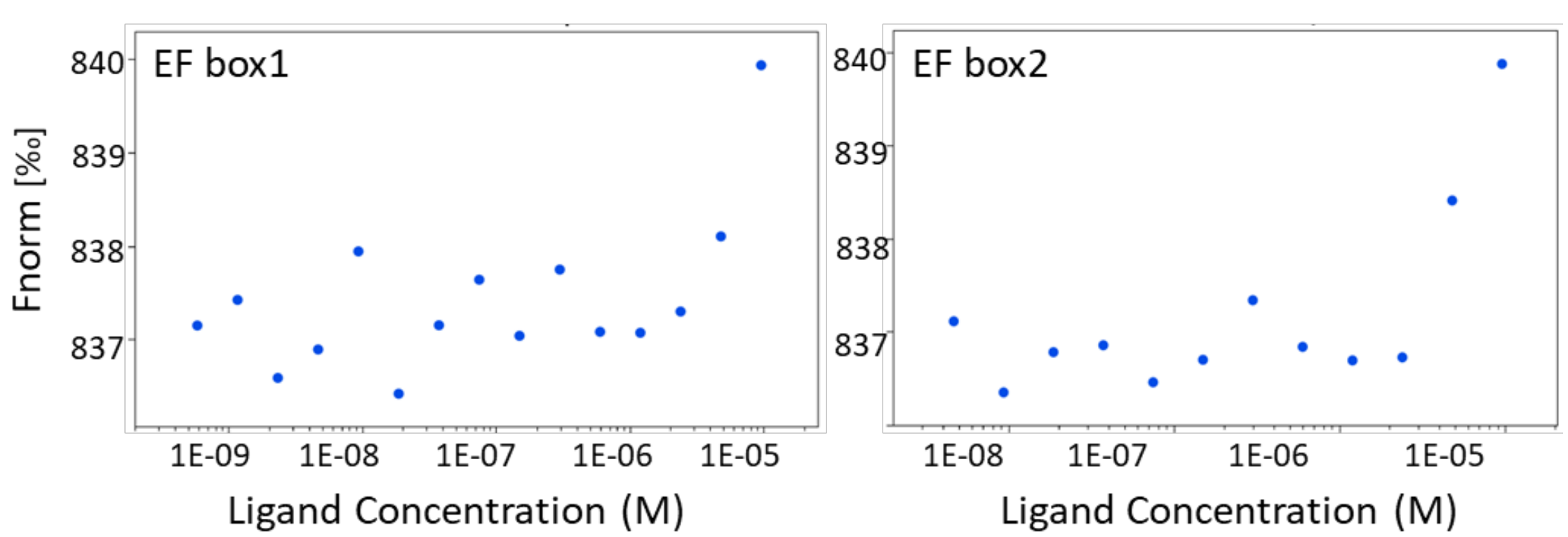

Figure A2. Binding of EcoNrdR to individual boxes of the *E. coli nrdEF* promoter. No significant binding was detected under the conditions tested.

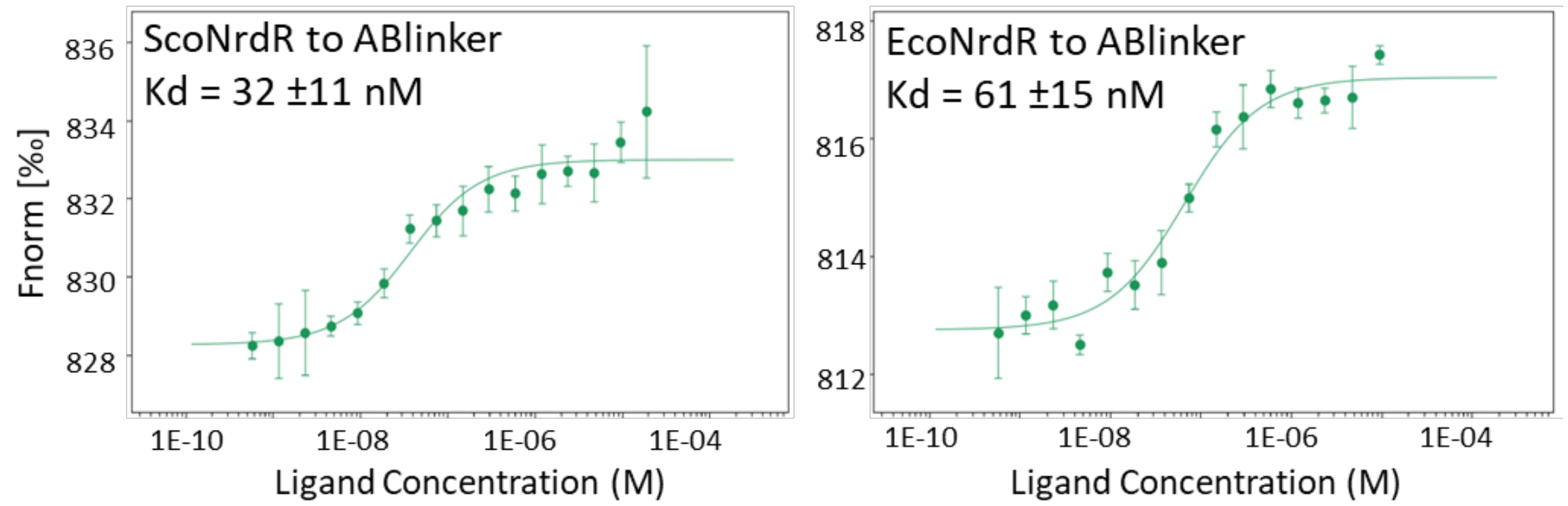

Figure A3. Binding of ScoNrdR and EcoNrdR to the Synt DNA fragment with native *S. coelicolor nrdAB* linker region.

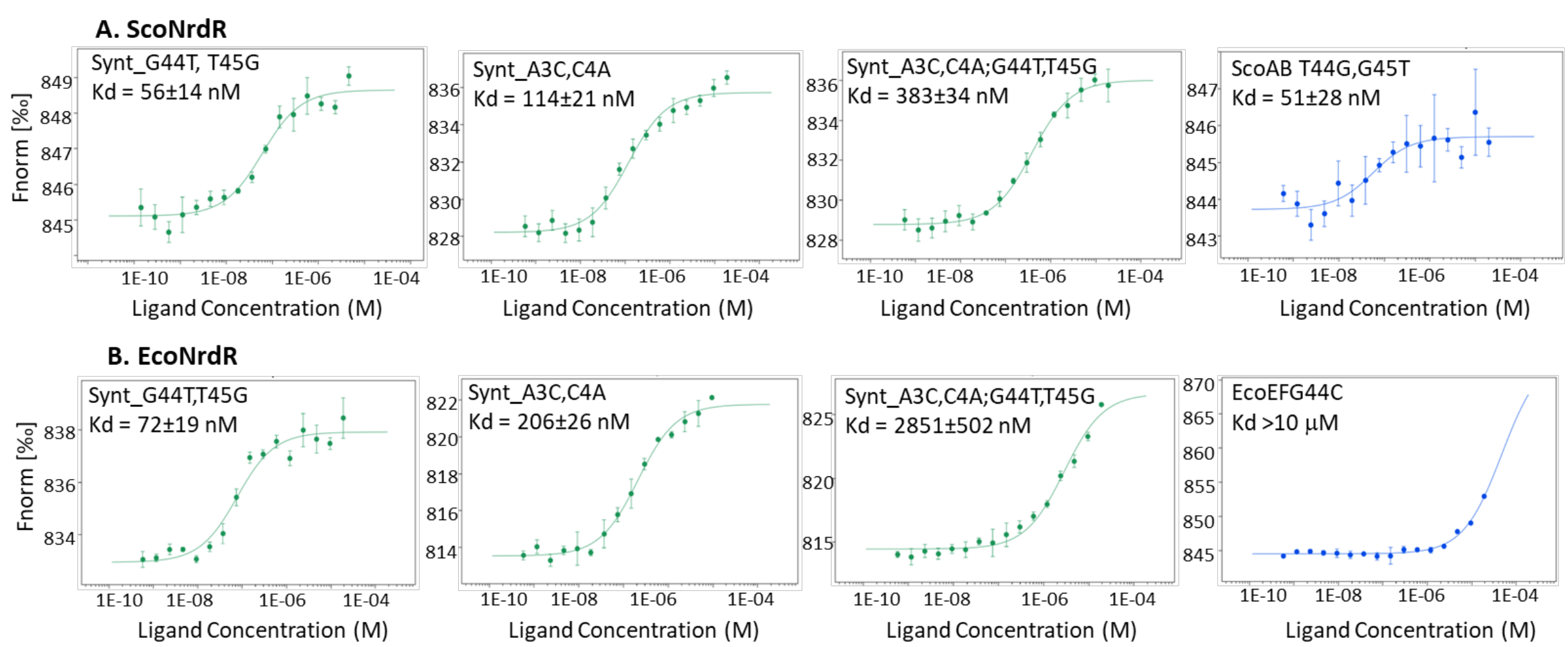

5'-gccaa**ACACAACATCTAGTGG**ttggatagcgtgagc**ACACAACATCTAGTGG**acctc-3'

4 **13** **35** **44**

Figure A4. Binding of ScoNrdR /A) and EcoNrdR (B) to mutated Synt DNA fragments and mutated RNR promoter regions. Green curves, binding to Synt DNA; blue curves, binding to corresponding mutations in homologous *S. coelicolor nrdAB* and *E. coli nrdEF* promoter regions, respectively. Lower line shows the sequence of a DNA fragment used with NrdR boxes in bold and mutated bp in red.

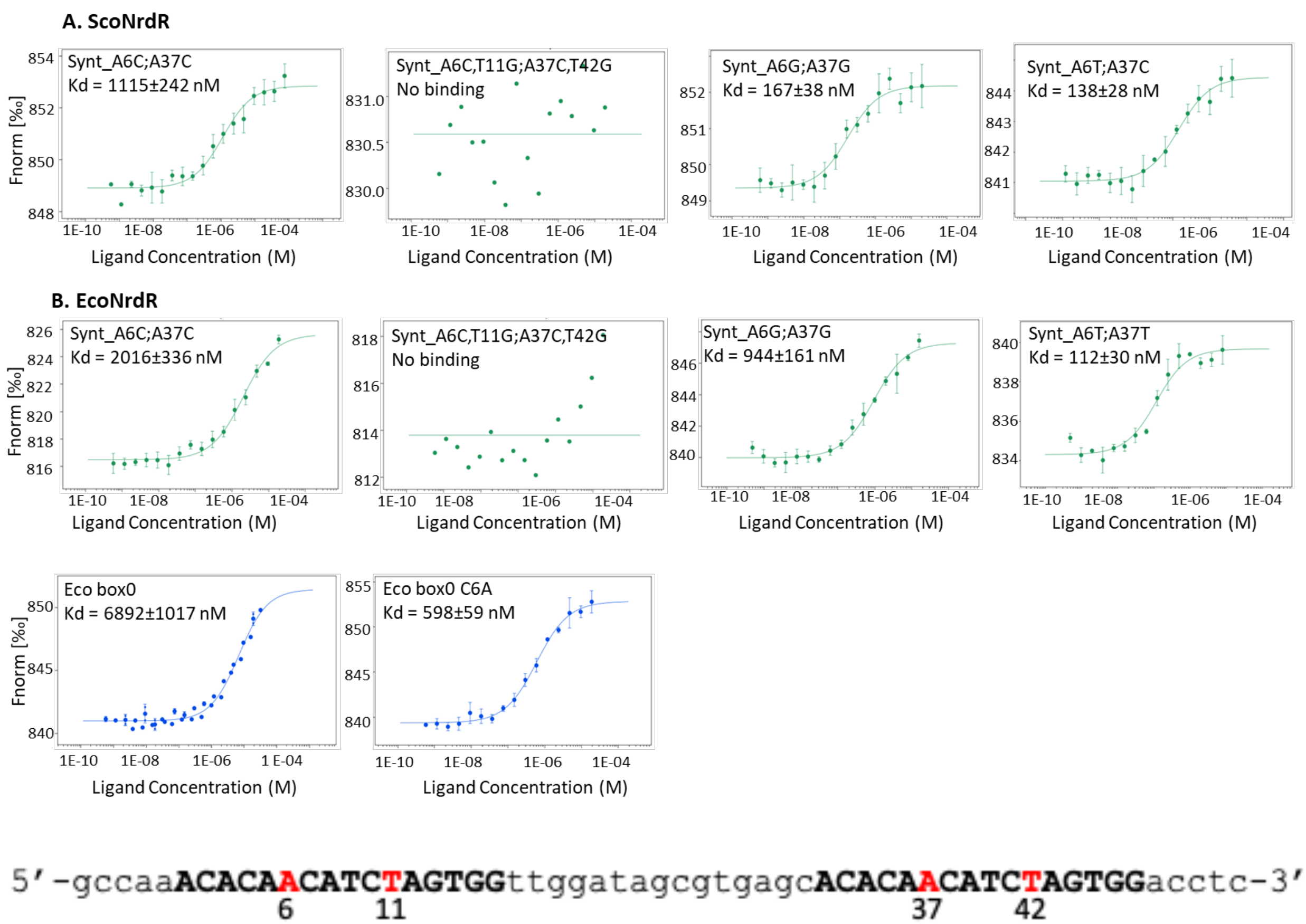

Figure A5. Binding of ScoNrdR (A) and EcoNrdR (B) to mutated Synt DNA fragments and *E. coli* RNR promoter regions. Green curves, binding to Synt DNA. Blue curves show EcoNrdR binding to native and mutated *E. coli nrdAB* promoter fragment comprising NrdR boxes 0 and 1. Lower line shows sequence of DNA fragment used with NrdR boxes in bold and mutated bp in red.

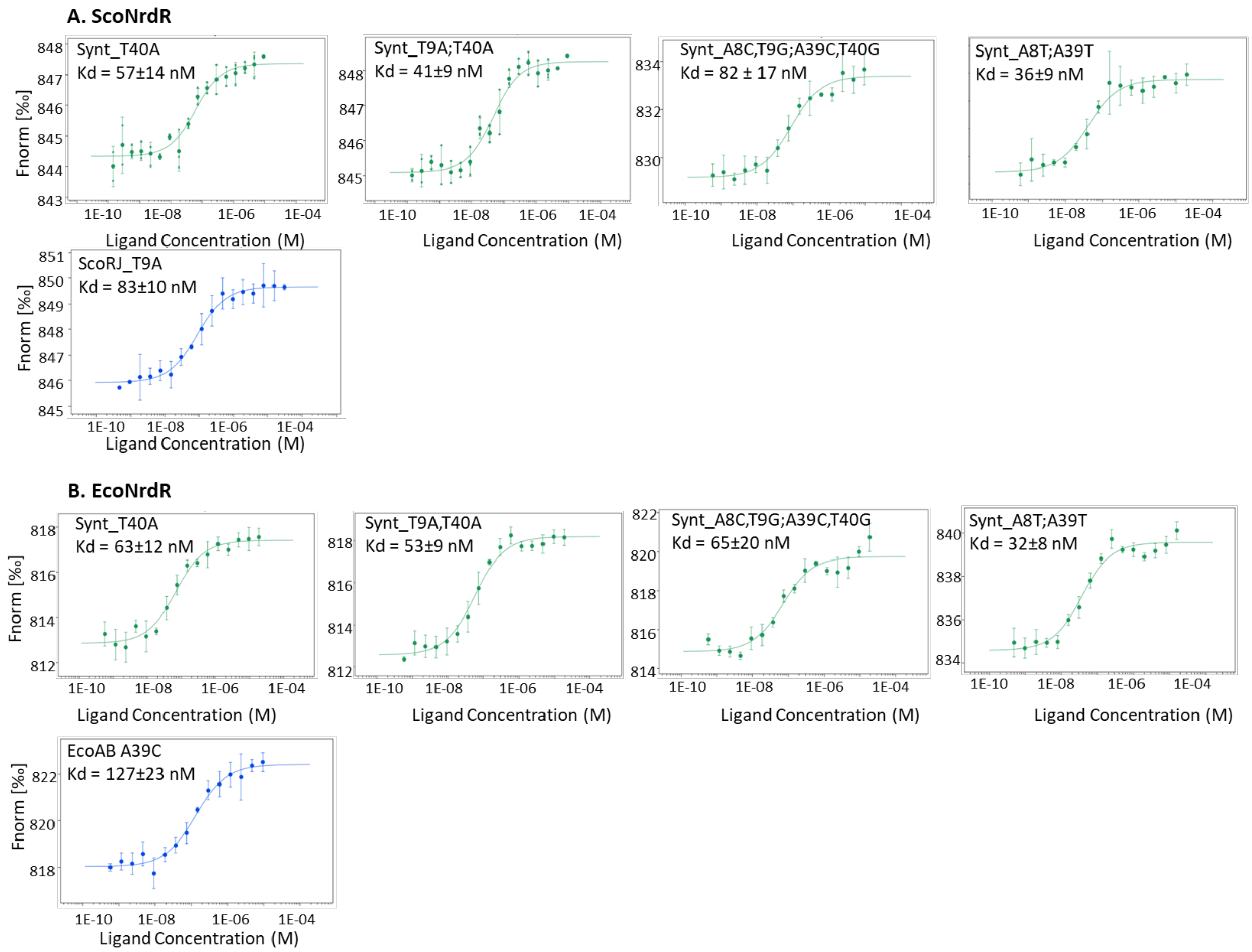

5'-gccaa**ACACAACATCTAGTGG**ttggatagcgtgagc**ACACAACATCTAGTGG**acctc-3'
8 9 39 40

Figure A6. Binding of ScoNrdR (A) and EcoNrdR (B) to mutated Synt DNA fragments and mutated RNR promoter regions. Green curves, binding to Synt DNA; blue curves, binding to corresponding mutations in homologous *S. coelicolor nrdRJ* and *E. coli nrdAB* promoter regions, respectively. Lower line shows sequence of DNA fragment used with NrdR boxes in bold and mutated bp in red.

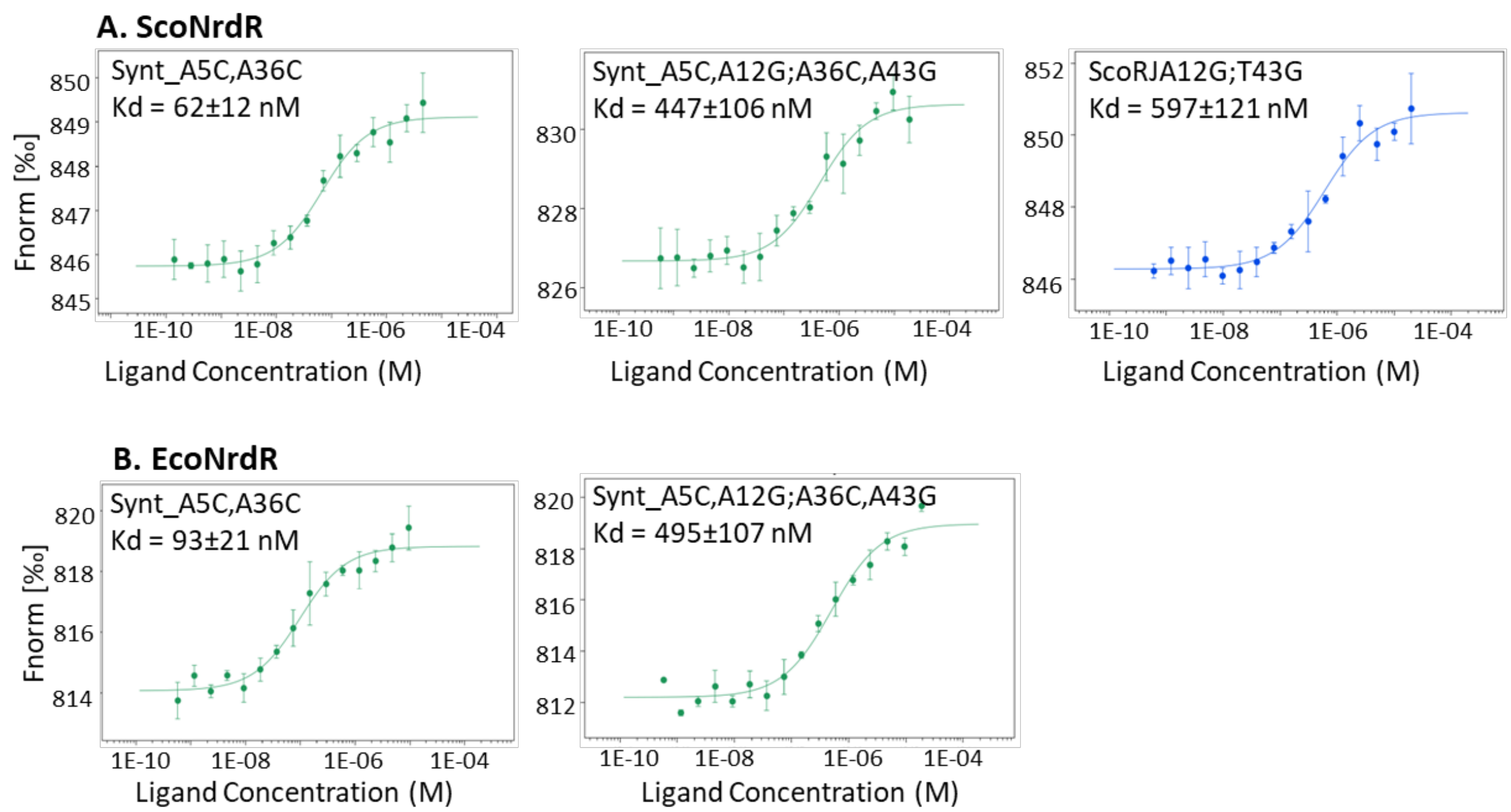

5′-gccaa**ACACAACATCTAGTGG**ttggatagcgtgagc**ACACAACATCTAGTGG**acctc-3′
5 12 36 43

Figure A7. Binding of ScoNrdR (A) and EcoNrdR (B) to mutated Synt DNA fragments and mutated *S. coelicolor* RNR promoter regions. Green curves, binding to Synt DNA; blue curve, binding to corresponding mutations in homologous *S. coelicolor nrdRJ* promoter region. Lower line shows sequence of DNA fragment used with NrdR boxes in bold and mutated bp in red.

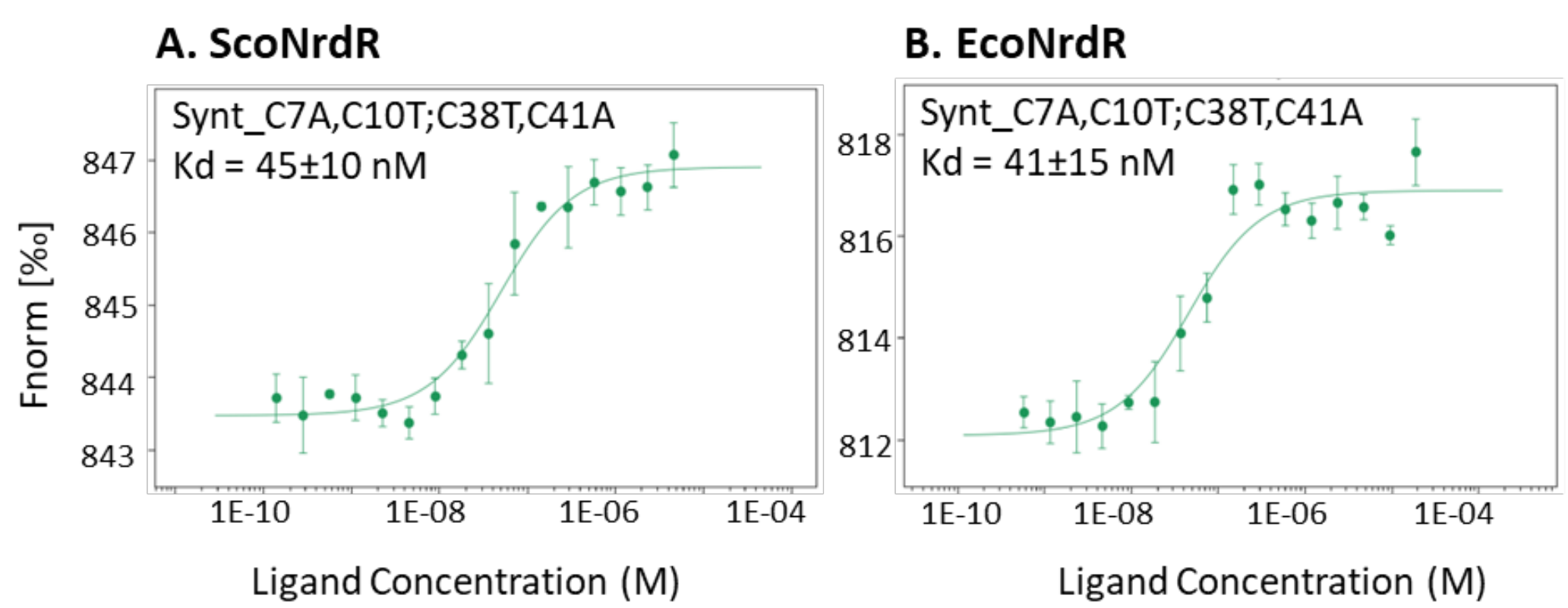

5′-gccaa**ACACAACATCTAGTGG**ttggatagcgtgagc**ACACAACATCTAGTGG**acctc-3′
7 10 38 41

Figure A8. Binding of ScoNrdR (A) and EcoNrdR (B) to mutated Synt DNA fragments. Green curves, binding to Synt DNA. Lower line shows the sequence of a DNA fragment used with NrdR boxes in bold and mutated bp in red.

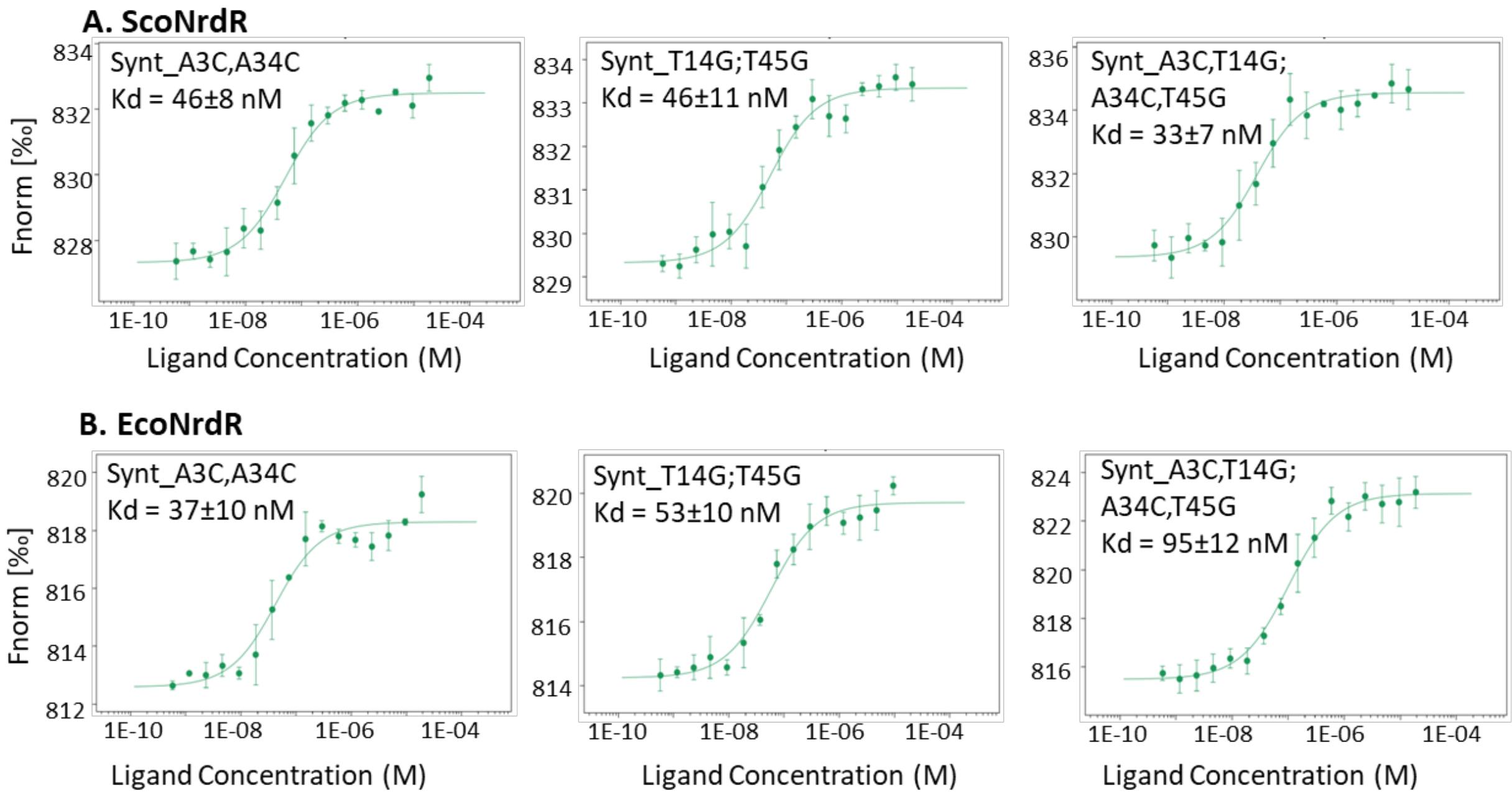

5'-gccaa**ACACAACATCTAGTGG**ttggatagcgtgagc**ACACAACATCTAGTGG**acctc-3'
3 14 34 45

Figure A9. Binding of ScoNrdR (A) and EcoNrdR (B) to mutated Synt DNA fragments. Lower line shows sequence of DNA fragment used with NrdR boxes in bold and mutated bp in red.

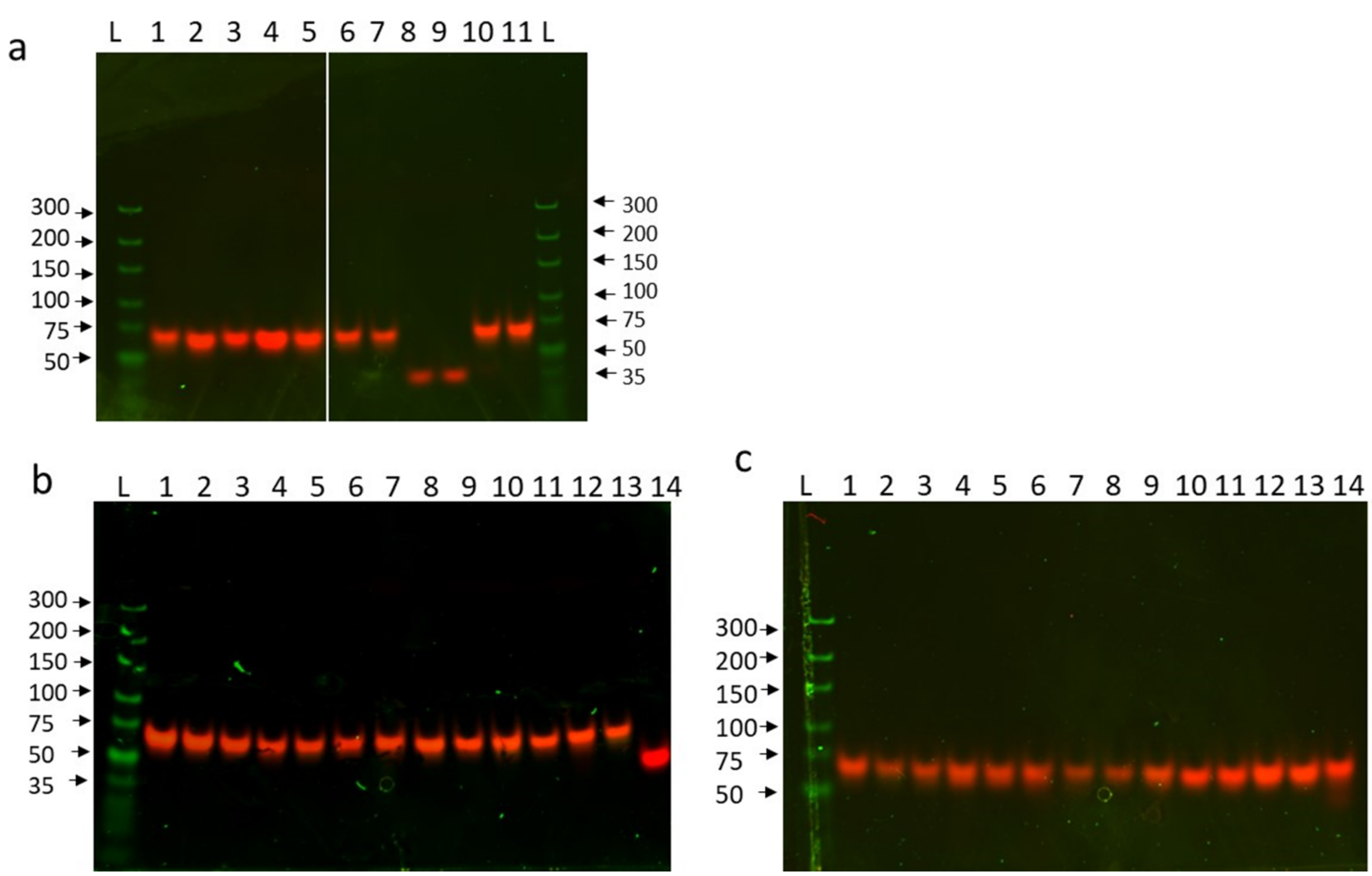

Figure A10. PAGE of annealed oligonucleotides used for MST. A: L) DNA ladder; 1) Eco nrdAB_0,1; 2) Eco nrdAB_1,2; 3) Eco nrdEF; 4) Eco nrdDG; 5) Eco RibX; 6) Eco nrdEF duplicated box1; 7) Eco nrdEF duplicated box2; 8) Eco nrdEF box1; 9) Eco nrdEF box2; 10) Sco nrdRJ; 11) Sco nrdAB. **B:** L) DNA ladder; 1) Synt 2) Synt T9A,T40A 3) Synt T40A; 4) Synt A8C,T9G,A39C,T40G; 5) Synt A8T,A39T; 6) Synt C7T,C10A,C38T,C41A 7) Synt A6C,A37C; 8) Synt A6C,T11G,A37C,T42G; 9) Synt A6T,A37T; 10) Synt A5C,A36C; 11) Synt A5C,A12G,A36C,A43G; 12) Synt A6T,A37T; 13) Synt ScoABlinker; 14) Synt_For. **C:** L) DNA ladder; 1) Synt A3C,C4A; 2) Synt G44T,T45G; 3) Synt A3C,C4A,G44T,T45G; 4) Synt T14G,T45G; 5) Synt A3C,A34C; 6) Synt A3C,T14G,A34C,T45G; 7) Sco nrdAB T44G G45T; 8) Sco nrdRJ T9A; 9) Sco nrdRJ A12G T43G ; 10) Eco nrdEF G44C; 11) Eco nrdEF T12G G13T; 12) Eco nrdAB0,1 C6A; 13) Eco AB1,2 A39C; 14) Synt. Double stranded oligonucleotide (1 pmol) was loaded on 5% polyacrylamide gel in Tris/Borate/EDTA (TBE) buffer and ran at 70 V for ~90 min. Gene ruler Ultra low (Thermo Fischer Scientific) was used as DNA ladder.

Table A1. List of oligonucleotides used in MST experiments. Additional oligonucleotides used in this study have been published[1-3].

MST oligonucleotides	ssDNA sequence
nrdEF_box1_ sense	5'-Cy5-GTCTCTTGCTATATATTGTGTCTCTG-3'
nrdEF_box1_antisense	5'-CAGAGACACAATATATAGCAAGAGAC-3'
nrdEF_box2_ sense	5'-Cy5-GTCTCCAACTACATCTAGTATCTCTG-3'
nrdEF_box2_antisense	5'-CAGAGATACTAGATGTAGTTGGAGAC-3'
Synt DNA_sense[4]	5'-Cy5-GCCAAACACAACATCTAGTGGTTGGATAGCGTGAGCACACAACATCTAGTGGACCTC-3'
Synt DNA_antisense[4]	5'-GAGGTCCACTAGATGTTGTGTGCTCACGCTATCCAACCACTAGATGTTGTGTTTGGC-3'
Synt DNA_SconrdABlinker_sense	5'-Cy5-GCCAAACACAACATCTAGTGGTGCTCGCGTCCCCCGACACAACATCTAGTGGACCTC-3'
Synt DNA_SconrdABlinker_antisense	5'-GAGGTCCACTAGATGTTGTGTCGGGGGACGCGAGCACCACTAGATGTTGTGTTTGGC-3'
Sco*nrdAB* T44G, G45T_sense	5'-Cy5-CCGGGACACAACATCTGGGGGTGCTCGCGTCCCCCGGCACAAGATGTAGTCTCATGC-3'
Sco*nrdAB* T44G, G45T_antisense	5'-GCATGAGACTACATCTTGTGCCGGGGGACGCGAGCACCCCCAGATGTTGTGTCCCGG-3'
Eco*nrdEF* G44C_sense	5′-Cy5-ACTATTTGCTATATATTGTGTGGTTGAATCTTTTTTCAACTACATCTACTATCTCTG-3′
Eco*nrdEF* G44C_antisense	5′-CAGAGATAGTAGATGTAGTTGAAAAAAGATTCAACCACACAATATATAGCAAATAGT-3′
Eco*nrdEF* T12G, G13T_sense	5′-Cy5-ACTATTTGCTATATATGTTGTGGTTGAATCTTTTTTCAACTACATCTAGTATCTCTG-3′
Eco*nrdEF* T12G, G13T_antisense	5′-CAGAGATACTAGATGTAGTTGAAAAAAGATTCAACCACAACATATATAGCAAATAGT-3′

Eco*nrdAB* box 0,1 C6A_sense	5′-Cy5-CATTTTCACAATATCTTGCAGTGAATCCCAAACATACCCCCTATATATAGTGTTCTAA-3′
Eco*nrdAB* box 0,1 C6A_antisense	5′-TTAGAACACTATATATAGGGGGTATGTTTGGGATTCACTGCAAGATATTGTGAAAATG-3′
Sco*nrdRJ* T9A_sense	5'-Cy5-GCCAATCCCCACAACTAGTGGTTGGATAGCGTGAGCAGCCCACAAGTTGTGGTCCCC-3'
Sco*nrdRJ* T9A_antisense	5′-GGGGACCACAACTTGTGGGCTCACGCTATCCAACCACTAGTTGTGGGGATTGGC-3′
Eco*nrdAB* A39C_sense	5′-Cy5-CATACCCCCTATATATAGTGTTCTAAGCAGCTTCCCGTACTACCGGTAGTCTGCATG-3′
Eco*nrdAB* A39C_antisense	5′-CATGCAGACTACCGGTAGTACGGGAAGCTGCTTAGAACACTATATATAGGGGGTATG-3′
Sco*nrdRJ* A12G, T43G_sense	5'-Cy5-GCCAATCCCCACATCTGGTGGTTGGATAGCGTGAGCAGCCCACAAGTGGTGGTCCCC-3'
Sco*nrdRJ* A12G,T43G_antisense	5′-GGGGACCACCACTTGTGGGCTCACGCTATCCAACCAACCAGATGTGGGGATTGGC-3′

[1]Rozman Grinberg I, Martínez-Carranza M, Bimaï O, Nouaïria G, Shahid S, Lundin D, Logan DT, Sjöberg BM, Stenmark P. 2022. A nucleotide-sensing oligomerization mechanism that controls NrdR-dependent transcription of ribonucleotide reductases. Nat Commun 13:2700.

[2]Rozman Grinberg I, Bimaï O, Shahid S, Philipp L, Martínez-Carranza M, Banerjee I, Lundin D, Stenmark P, Sjöberg B-M, Logan DT. 2025. Bacterial transcriptional repressor NrdR - a flexible multifactorial nucleotide sensor. FEBS J 292:3091-3112.

[3]Shahid S, Balka M, Lundin D, Daley DO, Sjöberg B-M, Rozman Grinberg I. 2025. NrdR in Streptococcus and Listeria spp.: DNA helix phase dependence of the bacterial ribonucleotide reductase repressor. Mol Microbiol 123:406-419.

[4]This corresponds to the Synt DNA with the SconrdRJ linker used in mutation experiments.
